# New Insights on the Mechanism of the K^+^-Independent Activity of Crenarchaeota Pyruvate Kinases

**DOI:** 10.1371/journal.pone.0119233

**Published:** 2015-03-26

**Authors:** Gustavo De la Vega-Ruíz, Lenin Domínguez-Ramírez, Héctor Riveros-Rosas, Carlos Guerrero-Mendiola, Alfredo Torres-Larios, Gloria Hernández-Alcántara, José J. García-Trejo, Leticia Ramírez-Silva

**Affiliations:** 1 Departamento de Bioquímica, Facultad de Medicina, Universidad Nacional Autónoma de México, 04510 Distrito Federal, México; 2 Departamento de Ciencias Químico-Biológicas, Universidad de las Américas-Puebla, Ex-Hacienda Santa Catarina Mártir, Cholula, 72820 Puebla, México; 3 Departamento de Bioquímica, Instituto de Fisiología Celular, Universidad Nacional Autónoma de México, 04510 Distrito Federal, México; 4 Departamento de Biología, Facultad de Química, Universidad Nacional Autónoma de México, 04510 Distrito Federal, México; University of Copenhagen, DENMARK

## Abstract

Eukarya pyruvate kinases have glutamate at position 117 (numbered according to the rabbit muscle enzyme), whereas in Bacteria have either glutamate or lysine and in Archaea have other residues. Glutamate at this position makes pyruvate kinases K^+^-dependent, whereas lysine confers K^+^-independence because the positively charged residue substitutes for the monovalent cation charge. Interestingly, pyruvate kinases from two characterized Crenarchaeota exhibit K^+^-independent activity, despite having serine at the equivalent position. To better understand pyruvate kinase catalytic activity in the absence of K^+^ or an internal positive charge, the *Thermofilum pendens* pyruvate kinase (valine at the equivalent position) was characterized. The enzyme activity was K^+^-independent. The kinetic mechanism was random order with a rapid equilibrium, which is equal to the mechanism of the rabbit muscle enzyme in the presence of K^+^ or the mutant E117K in the absence of K^+^. Thus, the substrate binding order of the *T*. *pendens* enzyme was independent despite lacking an internal positive charge. Thermal stability studies of this enzyme showed two calorimetric transitions, one attributable to the A and C domains (*T_m_* of 99.2°C), and the other (*T_m_* of 105.2°C) associated with the B domain. In contrast, the rabbit muscle enzyme exhibits a single calorimetric transition (*T_m_* of 65.2°C). The calorimetric and kinetic data indicate that the B domain of this hyperthermophilic enzyme is more stable than the rest of the protein with a conformation that induces the catalytic readiness of the enzyme. B domain interactions of pyruvate kinases that have been determined in *Pyrobaculum aerophilum* and modeled in *T*. *pendens* were compared with those of the rabbit muscle enzyme. The results show that intra- and interdomain interactions of the Crenarchaeota enzymes may account for their higher B domain stability. Thus the structural arrangement of the *T*. *pendens* pyruvate kinase could allow charge-independent catalysis.

## Introduction

Rabbit muscle pyruvate kinase (RMPK) was the first enzyme reported to have an absolute requirement for K^+^ [1]. Despite extensive study [2,3,4,5,6], the role of the K^+^ in catalysis by RMPK is not yet completely understood. Recently, it has been proposed that the K^+^ is directly involved in the movement of the active site lid (B domain) during the transition of PK to its active conformation. This conformation allows either phosphoenolpyruvate (PEP) or ADP to bind, following a random-order kinetic mechanism [7]. For a long time, it was thought that the dependence on K^+^ was a feature common to all PKs [8]. However, as more enzymes were characterized, it became apparent that the activity of many PKs is K^+^ independent [8]. To explore the molecular basis underlying this behavior, Laughlin and Reed [9] compared the amino acid sequence of RMPK with those of two K^+^-independent bacterial enzymes. These authors found that Glu117 of RMPK, which is close to the K^+^-binding site, was replaced by Lys in the bacterial enzymes. They constructed the E117K mutant of the rabbit enzyme and found that the mutant was not stimulated by monovalent cations. The authors proposed that the expression of the K^+^-independent activity was due to the internal positive charge supplied by the protonated Lys [9]. To determine the abundance of K^+^-independent PK enzymes, an extensive phylogenetic study of this enzyme family was performed [8]. Of the 230 sequences investigated, 121 contain a Glu at position 117 (according to RMPK numbering), 106 contain a Lys, 2 contain a Ser and 1 has an Arg at this position. The PKs containing a Glu and those containing a Lys are clearly separated into two clusters. All characterized members of the cluster with a Glu residue at position 117 exhibit K^+^-dependent activity, whereas those with a Lys residue exhibit K^+^-independent activity. The presence of Leu113/Gln114 and a hydrophobic residue (Ile, Leu, Val) at position 120 are covariant in 77% of the PKs that contain Lys117. These residues are replaced by Glu117/Thr113/Lys114/Thr120 in 80% of the K^+^-dependent PKs.

Laughlin and Reed [9] also constructed an E117A mutant of RMPK and found that, like the wild-type, its activity was absolutely dependent on K^+^. This result confirmed their hypothesis that the enzyme requires an internal positive charge or K^+^ for catalytic activity. However, the PKs from *Pyrobaculum aerophilum* (*Pa*PK) and *Thermoproteus tenax* are K^+^-independent even if a non-positively charged residue (Ser) is located at the position that corresponds to residue 117 of RMPK [10,11]. These two homologs are located in the K^+^-independent branch of the phylogenetic tree of PKs [8] and belong to the Crenarchaeota subdomain. The phylogenetic analysis previously described, included only 18 sequences of the PK proteins from Archaea, and the number of sequences of PKs available is substantially larger today. In contrast to the conserved Glu117/Lys117 present in all PKs from the Bacteria and Eukarya domains, other amino acids are found in the corresponding position in the enzymes from the Crenarchaeota subdomain. In this work, an updated sequence alignment of the Archaeal PKs is presented (S1 Fig.). The previously observed co-evolution between residue 117 and residues 113, 114, and 120 [8] was not observed in the sequences of PKs from hyperthermophilic Crenarchaeota species, nor were the residues of the K^+^ binding site strictly conserved.

These data suggest that the K^+^-independent activity in Crenarchaeota PKs may result from a mechanism other than that involving the internal positive charge supplied by the protonated ε-amino group of Lys [9]. To further investigate these hyperthermophilic enzymes, we performed a new phylogenetic analysis, evaluated the kinetic properties, thermal stability, and molecular dynamics, and performed structural modeling of the pyruvate kinase from *Thermofilum pendens* (*Tp*PK), which has a hydrophobic residue at the position corresponding to the RMPK 117 (Val70). The results indicate that for *Tp*PK, the closure of the active site and the arrangement of the residues involved in the binding of the nucleotide are independent of the presence of an internal positive charge or K^+^ and may be related to the stability of the active site cleft.

## Materials and Methods

### Reagents

The endonucleases and T4 DNA ligase were purchased from New England Biolabs (UK). Platinum Taq DNA polymerase was from Invitrogen Life Technologies (MA, USA). Shrimp alkaline phosphatase was from Roche Applied Science. Miniprep plasmid purification kits were from QIAGEN (N.V.). The BL21 CodonPlus-pLysS strain of *E*. *coli*, the pMCSG7, pET-3a vectors and all oligonucleotides were from Invitrogen. RMPK and hog muscle lactate dehydrogenase (LDH) were obtained from Roche Applied Science (Mannheim, Germany). Antibiotics (ampicillin, chloramphenicol), Mes, Tris and the cyclohexylammonium salts of ADP and PEP (phosphoenolpyruvate) were from Sigma-Aldrich Co. (USA) NADH sodium salt was converted to the cyclohexylammonium salt by ion exchange following the protocol provided by the manufacturer (Sigma-Aldrich).

### Sequence analyses

The pyruvate kinase amino acid sequences from Archaea were retrieved by BlastP searches at the UniProt site [12] (http://www.uniprot.org). Progressive multiple amino acid sequence alignments were performed with ClustalX version 2 [13] (http://www.clustal.org/clustal2/) using a structural alignment constructed with the VAST algorithm as a guide [14]. The alignment included all non-redundant PK protein structures deposited in the Protein Data Bank [15]. Non-redundant protein sequences from a previous phylogenetic analysis [8] were included in the multiple-sequence alignment and corrected manually using BioEdit [16] (http://www.mbio.ncsu.edu/bioedit/bioedit.html). Phylogenetic analyses were conducted using the MEGA5 software [17] (http://www.megasoftware.net). Four methods were used to infer the phylogenetic relationships: maximum likelihood, maximum parsimony, minimum evolution, and neighbor joining. The amino acid substitution model described by Whelan and Goldman [18] using a discrete Gamma distribution with five categories was chosen as the best substitution model because it gave the lowest Bayesian Information Criterion values and corrected Akaike Information Criterion values [19] in MEGA5 [17]. The gamma shape parameter value (+G parameter = 0.92) was estimated directly from the data with MEGA5. The confidence for the internal branches of the phylogenetic tree, obtained using the maximum likelihood method, was determined through bootstrap analysis (500 replicates each).

Sequence logos were constructed using the WebLogo server (http://weblogo.threeplusone.com/). Each logo consists of stacks of amino acid letters. The ordinate axis of the logos graphs, indicate the stack for each position in the sequence. The height of the letters within the stack indicates the relative frequency of each amino acid at that position [20].

### Cloning of *Tp*PK

The PK gene from *T*. *pendens* was amplified from the genomic DNA of the strain Hrk. The gene was amplified by PCR using the primers FW 5′-TACTTCCAATCC AATGCTGCAAAAGTCAAGCTAGTAGCGCG-3′ and RV 5′-TTATCCACTTCCAATGTT ACTCGCTCTTATCTCTCCACGG-3′. The insert was introduced into the pMCSG7 vector, a pET-based expression vector that has a His_6_ tag at the N-terminus and a Tobacco Etch Virus (TEV) protease cleavage site, by ligation-independent cloning using published protocols [21]. The vector was treated with *Ssp*I followed by T4 DNA polymerase in the presence of dGTP, and the PCR product was treated with polymerase in the presence of dCTP. After annealing, freshly prepared competent *Escherichia coli* XL-Gold were transformed with the plasmids. The plasmids were isolated and sequenced to verify the absence of mutations.

### Cloning of the B domain

The fragment encoding the B domain was amplified from the *Tp*PK plasmid by PCR, using the following primers FW 5′-TACCATATGAGGCTTGGAGAG-3′ and the RV 5′-ATTGGATCCGACGGTCACAGT-3′. The PCR product was cloned into the pET-3a vector using two restriction sites (Nde1 and BamH1). The pET-3a vector was transformed into *E*. *coli* XL-Gold. The plasmids were isolated and sequenced to verify the absence of mutations.

### Cell growth and purification of *Tp*PK

LB medium containing 100 μg/ml ampicillin and 34 μg/ml chloramphenicol was inoculated with BL21 cells containing the plasmid CodonPlus-pLysS-*Tp*PK at 37°C at an *A*_600_ of approximately 0.4. Expression was induced overnight with 0.5 mM isopropyl 1-thio-β-D-galactopyranoside at 15°C. Recombinant *E*. *coli* cells were suspended in 50 mM KH_2_PO_4_ pH 8, 10 mM Imidazol, 300 mM KCl and half of a tablet of Complete Protease Inhibitors (Roche Applied Science). The cells were lysed by sonication with a Sonifier 450 (Branson) for 2.5 min at 40 kHz. The suspension was centrifuged, the supernatant was heated at 80°C for 30 min, and the precipitated protein was discarded. The supernatant was loaded on a His Trap FF column, and the enzyme was eluted with a linear gradient of imidazole (10–500 mM). The fractions that exhibited PK activity (~ 250 mM imidazole) were pooled and concentrated by membrane filtration (Centricon 30,000 MWT) and then desalted on a Hi Trap Desalting column. This pool was incubated for 48 h with Tobacco Etch Virus (TEV) protease at a ratio 1:30 (*Tp*PK: TEV). The enzyme was then loaded on a His Trap column. The fractions with activity were pooled and concentrated. In this step, less than 0.5% of the *Tp*PK was recovered. Therefore, in subsequent purifications, the incubation with TEV protease was omitted. The enzyme that was still bound to the His Trap FF column was eluted with a linear gradient of imidazole. The fractions that exhibited PK activity were pooled, concentrated (Centricon 30,000 MWT) and desalted on a Hi Trap Desalting column. This pool was loaded on a DEAE Sepharose column, and the enzyme was eluted with a linear gradient of KCl (0–1 M). The purity of the *Tp*PK that eluted from the imidazole gradient was 89% (in this step, the purity of *Tp*PK with or without His_6_ tag was similar; gel not shown), whereas that of the enzyme after the anionic-exchange step was >95% as determined by SDS-PAGE (12.5%). Mass spectrometric characterization of the protein by ESI-MS was performed at the Research Resources Center at the University of Illinois, Chicago (spectrum not shown). The enzyme was precipitated with ammonium sulfate at 80% saturation and stored at 4°C. To determine the oligomerization state of the enzyme, BN-PAGE was carried out overnight at 4°C as described [22] using 150 μg of *Tp*PK.

### Cell growth and purification of the B domain

LB medium containing 100 μg/ml ampicillin and 34 μg/ml chloramphenicol was inoculated with BL21 cells containing the plasmid CodonPlus-pLysS-B domain at 37°C at an *A*_600_ of approximately 0.4. Expression was induced with 0.5 mM isopropyl 1-thio-β-D-galactopyranoside at 20°C, and the cells were harvested after overnight growth. Recombinant *E*. *coli* cells were suspended in 50 mM Tris-HCl pH 7.0 containing half of a tablet of Complete Protease Inhibitors (Roche Applied Science). The cells were lysed by sonication with a Sonifier 450 (Branson) for 2.5 min at 40 kHz. The suspension was centrifuged 30 min at 20,000 X *g*. The supernatant was precipitated with ammonium sulfate at 37% saturation, and the resultant supernatant was collected. A second precipitation with ammonium sulfate at 80% saturation was performed. The pellet was collected, suspended and desalted by dialysis. The final steps involved ion exchange chromatography using DEAE and molecular exclusion using Superdex 75. SDS-PAGE was performed, and the fractions with MW ~10,000 Da were pooled and concentrated. The B domain was 95% pure, as indicated by SDS-PAGE (not shown).

### Mass spectroscopy of the B domain of *Tp*PK

The protein band with the expected molecular weight of the B domain was excised from the SDS-PAGE gel. The sample was digested in-gel with trypsin and was then injected into an integrated nano-LC-ESI-MS/MS system (quadrupole/time of flight, Ultima API, Micromass, Manchester, UK). The acquired peptide ions were analyzed with the Mascot program (www.matrixscience.com) using both the NCBInr and the EST databases. Only proteins with significant ion scores (> 46) were reported. The sequence of all peptides matched the B domain of *Tp*PK (data not shown). These experiments were performed by Dr. Maire Christine Slommiary of Université des Sciences at Technologies de Lille, Villeneuve d′ Ascq, France.

### Assays of pyruvate kinase activity

LDH was obtained as an ammonium sulfate suspension from Roche Applied Science. Ammonium sulfate-free enzymes were obtained as described previously [23]. Contaminating NH_4_^+^, Na^+^, and K^+^ in the reaction mixtures were below the detection limit (10 μM), as indicated elsewhere [24]. The formation of pyruvate was measured at 45°C in a coupled system with LDH and NADH [25]. The reaction mixtures contained 50 mM Mes-Tris, pH 6.0 and the indicated concentrations of cations (Mg^2+^ and Mn^2+^), substrates (PEP and ADP), and inhibitors (oxalate and AMP). The ADP-Mg complexes and free Mg^2+^ concentrations were calculated using the software CHELATOR [26]. The ADP-Mn complexes and free Mn^2+^ concentrations were calculated using the *K*_*d*_ of Mn^2+^ [27]. The ionized PEP concentrations were calculated considering a p*K* value of 6.3 [28]. (CH_3_)_4_NCl was added to a final salt concentration of 0.25 M to compensate for the different ligand (PEP, ADP-Mg, ADP-Mn, free divalent cations) concentrations. In the inhibition assays, the concentration of LDH added to the reaction mixture was sufficient to overcome the inhibition by oxalate. The specific activity was not increased by the inclusion of 5-fold higher concentrations of LDH. The reaction mixture was incubated for 10 min to reach the desired temperature (45°C) prior to initiating the reaction with *Tp*PK.

### Kinetic studies

The initial velocities of *Tp*PK activity were determined in the absence or presence of dead-end inhibitors (oxalate or AMP). In the former condition, the velocity patterns were obtained at various concentrations of PEP at several fixed concentrations of ADP-Mg. In the latter condition, the inhibition patterns were obtained by varying the concentration of one substrate with the second substrate fixed and at different fixed concentrations of the inhibitor.

### Differential scanning calorimetry (DSC)

All experiments were performed in a capillary Differential Scanning Microcalorimeter from GE Health Science (USA). The protein solutions were prepared in 50 mM Tris-HCl pH 7.6. The buffer in which the enzyme was prepared was placed in the reference cell. Unless otherwise indicated, the protein concentration was 1 mg/ml and the scan rate was 1.5°C/min. The experiments were conducted at temperatures ranging from 25 to 120°C.

### Models and structural analysis

The structures for RMPK (PDBID 2G50 was used as the open form, and 1A5U was used as the closed form) and *Pa*PK (PDBID 3QTG) were obtained from the PDB database (RCSB). The structures of *Tp*PK and the mutant F89I/F108I/F109C/F127L of *Tp*PK were modeled with Modeller version 9.2 [29] using the structure of *Pa*PK (the only hyperthermophilic PK structure from *Crenarchaeota* phylum that has been determined) as the template [30]: the identity between the two sequences was 30.6%. The model of the structure of *Tp*PK was validated with Molprobity (S1 Table) [31]. H-bonds were quantified using the Find H-Bond module in UCSF Chimera version 1.8.1 [32], and salt bridges were counted by first selecting the atoms involved in this type of interaction (Ng+, N3+, N2+, O2-, O3-) and then counting the interactions using the same Find H-bond module. π-π and π-cation interactions were counted using Yasara version 18.9.8 (“Yet Another Scientific Artificial Reality Application,” http://www.yasara.com) with the view interactions module. Specific interactions between domain A and domain B were identified by using Dimplot 4.5.3 [33] and its domain-domain interface module with the following definitions for each domain: domain B 66–164 (*Tp*PK), 81–177 (*Pa*PK) and 116–223 (RMPK); domain A 1–65 and 165–334 (*Tp*PK), 1–80 and 178–346 (*Pa*PK) and 43–115 and 224–387 (RMPK). Molecular dynamic simulations were run using AMBER12 [34]. The models were prepared with tleap using the AMBER FF12SB force-field and an implicit solvent model. Briefly, the system was first energy-minimized with 5000 steps of steepest descent followed by 5000 steps of conjugate gradients. Then, the system temperature was raised from 0 to the final simulation temperature (300, 400 or 500 K) over 200 ps. The production simulations (50 ns) were started after this step. For all simulation steps, a Langevin thermostat was used with a collision frequency of 1 per picosecond, and SHAKE was applied only to the hydrogen atoms with a step time of 2 femtoseconds. All simulations were run on GPUs [35]. Analysis of the native contacts was calculated using Carma [36,37].

## Results and Discussion

### Phylogenetic analysis of PKs

An updated bioinformatic analysis of PK [8] that included 191 new protein sequences from Archaea was performed. The new analysis consisted of 426 non-redundant PK sequences: 71 from Eukarya, 151 from Bacteria and 204 from Archaea. It is noteworthy that, without exception, the PKs from Archaea corresponded to single-copy genes. S1 Fig. shows that the PK protein sequences are divided into two main clusters: those with Glu at position 117 (according to RMPK numbering), all of which are K^+^- dependent, and those that mainly contain Lys at the corresponding position, all of which are K^+^- independent [8]. The analysis of the logos shows that position 117 covaries with positions 113, 114 and 120 (S1 Fig.).

**Fig 1:**
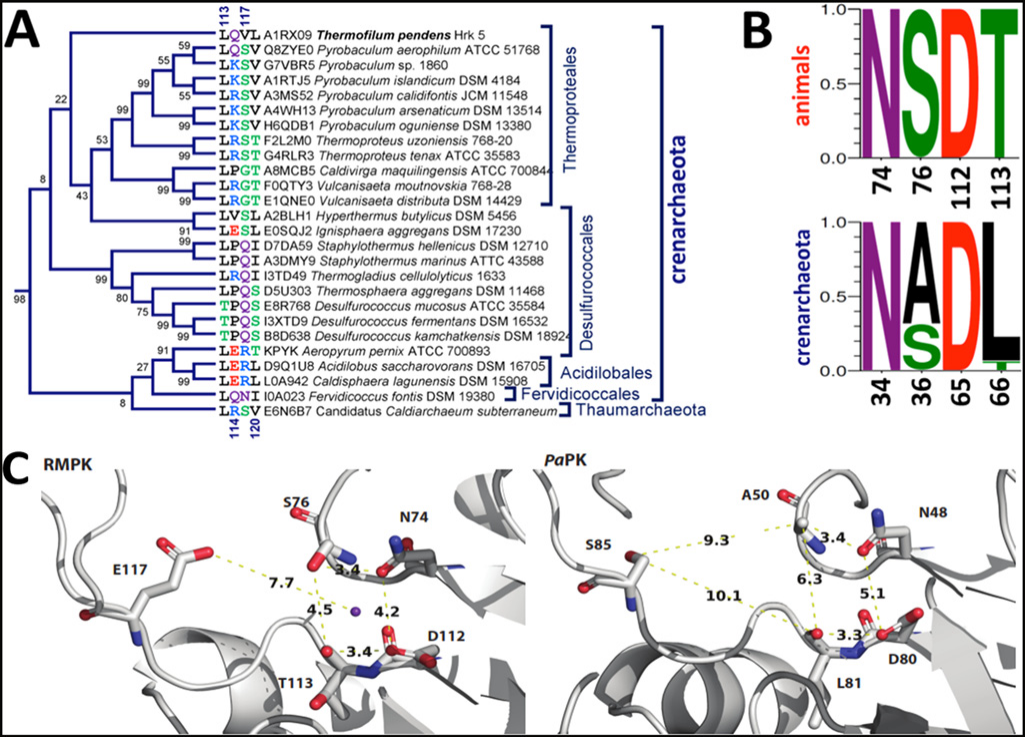
Conservation of the K^+^ binding site residues and of those that covariate with the corresponding position 117 in Crenarchaeota subdomain. In **A**. the subtree of the Crenarchaeota branch that contains the *Tp*PK sequence is shown. The unrooted phylogenetic tree that includes all available PK protein sequences from the Archaea domain is shown in Supplemental data (S1 Fig.): here, only the subtree of the Crenarchaeota branch that included *Tp*PK is presented. Residues 113, 114, 117, and 120 (according to RMPK numbering), as well as the accession numbers are indicated. The branches are not drawn to scale, and only the branch topology is shown. **B**. Logos of the K^+^ binding site residues in PK from animals and from the Crenarchaeota subdomain are presented. In animals, the numbering is according to RMPK, and the equivalent positions in the Crenarchaeota subdomain are given according to *Tp*PK numbering. **C**. The K^+^ binding site in RMPK (PDBID 1A49, subunit B) and hypothetical residues of coordination to the monovalent cation in *Pa*PK (PDBID 3QTG). In RMPK, K^+^ is shown in purple and the distances from K^+^ to Oγ of Ser76, O∂1 of Asn74, O∂1 of Asp112 and the carbonyl oxygen of Thr113 are 3.1, 2.6, 2.6 and 2.8 Å, respectively (not shown). The distances shown are those between the coordination residues and from O∂2 of Glu117 to K^+^ (RMPK) or from Oγ of Ser85 to Cß of Ala50 and the carbonyl oxygen of Leu81 (*Pa*PK).

The *Tp*PK sequence is clustered with the other Crenarchaeota PKs in a branch that includes PK sequences from thermoproteales, desulfurococcales, acidilobales and fervidicoccales (Fig. 1A). This branch is located within the cluster that comprises the K^+^-independent enzymes (see S1 Fig.). However, this branch contains the sequences of PKs in which residues other than Lys at position 117 are present (Ser, Gln, Arg, Asn, Gly and Val) (Fig. 1A and S1 Fig.). For instance, *Tp*PK has Val70 at the equivalent position, whereas the PKs from other Crenarchaeota species, such as *Pyrobaculum aerophilum* and *Thermoproteus tenax*, have Ser at this position and are also K^+^-independent [10,11]. This finding suggests that catalysis by these enzymes utilizes a mechanism other than that of an internal positive charge provided by Lys. Interestingly, in this Crenarchaeota branch, the covariation of the residues at positions 114, 117 and 120 is not conserved. In contrast, *Pa*PK and *Tp*PK are the only two Crenarchaeota PKs that share the covariation of residues at positions 113, 114 and 120 with the K^+^-independent enzymes. The other sequences of the PKs in this unusual Crenarchaeota branch share only one or two residues of the K^+^-independent signature. As described in [8], the residues that form the K^+^ binding site are highly conserved either in the K^+^-dependent or-independent PK branches, with the exception of Thr113, which is almost exclusively observed in the K^+^-dependent PKs. In the Crenarchaeota branch, only two of the four oxygens that putatively coordinate with the K^+^ are conserved (Oδ1 of Asn34 and Oδ2 of Asp65), and only 30% of the sequences contain the third one (Oγ of Ser36) (Fig. 1B). This result indicates that these residues are not sufficient to coordinate the K^+^. A comparison of the K^+^-binding site in RMPK with the putative monovalent site in *Pa*PK is shown in Fig. 1C. Remarkably, the distances between the oxygen atoms of the residues involved in the coordination sphere of K^+^ in RMPK are shorter than those found in the corresponding residues in *Pa*PK. Moreover, the distance between O∂2 of Glu117 and Oγ of Ser76 is 5.1 Å in RMPK, whereas the distance between Oγ of Ser85 and Cß of Ala50 is 9.3 Å in *Pa*PK. This observation indicates that Glu117 is close to the K^+^-binding site and is a key residue in the dependence of RMPK on monovalent cations. In contrast, in *Pa*PK, this position (Ser85) is probably too far away to affect this site. It is relevant to consider that the rotation angle of the B domain relative to the A domain is 41° for RMPK (PDBID 1A49, B subunit). This subunit exhibits the most open cleft conformation reported for RMPK [38]; the distance from O∂2 of Glu117 to Oγ of Ser76 is 5.1 Å, and the distance to the carbonyl oxygen of Thr113 is 7.7 Å (not shown). These distances are shorter than the distances shown for *Pa*PK in Fig. 1C. Although a strict comparison between these two crystal structures (RMPK vs. *Pa*PK) cannot be made due to the lack of information concerning the rotation angle of the B domain over the A domain for *Pa*PK (PDBID 3QTG) [30], Ser cannot occupy the same site that Glu does due to the differences in the sizes of the two residues.

### Purification of *Tp*PK and molecular mass determination

*Tp*PK was purified as described previously (Material and Methods) and incubated in the presence of TEV protease; however, the recovery of the total *Tp*PK without the His_6_ was less than 0.5%. Therefore, unless otherwise indicated, the experiments were performed with the enzyme containing the 23 additional residues. In this preparation, a single Coomassie-stained band with an apparent molecular mass of 50 kDa was observed by SDS-PAGE, confirming its high degree of purity (S2A Fig.). The oligomeric state of *Tp*PK was determined by BN-PAGE [22], which showed a native band of approximately 200 kDa (S2B Fig.), indicating that like most of the known PKs, the *Tp*PK is a homotetramer [39]. Mass spectrometry of the enzyme yielded a molecular weight of 53,965 Da, with 51,340 Da corresponding to the monomer of *Tp*PK and 2,624.7 Da corresponding to the His_6_ tag and the cleavage site of protease TEV (data not shown).

### Catalytic Properties of *Tp*PK

Because there was no previous information available about *Tp*PK, the enzyme was characterized biochemically. The kinetic constants for ADP-Mg of *Tp*PK with and without His_6_ tag were similar (*Km* of 0.092 ± 0.012 mM and 0.14 ± 0.013 mM, and *V*_max_ of 175 ± 6 μmol/min mg and 176 ± 5 μmol/min mg without and with the His_6_ tag, respectively), ruling out any putative effect of the 23 extra residues on the kinetic behavior of *Tp*PK.

### Effect of monovalent cations

To date, three PKs from Crenarchaeota species (*Pyrobaculum aerophilum*, *Thermoproteus tenax* and *Aeropyrum pernix*) have been characterized [10,11]. The first two contain Ser85 and Ser69, respectively (corresponding to position 117 in RMPK), the last contains Arg72, and these enzymes express monovalent cation-independent activity. Consistent with the PKs from *Pyrobaculum aerophilum* and *Thermoproteus tenax*, *Tp*PK that has a non- positively charged residue at the position corresponding to residue 117 and exhibited K^+^-independent activity as indicated by absence of an increase in activity when monovalent cations were included in the reaction mixture (data not shown). The lack of activation by monovalent cations has been reported in all K^+^-independent enzymes, including those with Lys [40,41,42,43,44,45,46], Ser [10,11] or Arg [11] at the position corresponding to residue 117.

### Kinetics of *Tp*PK in the presence of Mg^2+^ and Mn^2+^

The effects of divalent metal ions on the catalytic activity of *Tp*PK were studied because divalent cations are essential for phosphate transfer [47]. Therefore, in the absence of monovalent cations, we explored the effect of the “ancient Mn^2+”^ [48] and of Mg^2+^, the physiologically relevant divalent metal ion. The kinetic constants in the presence of these two divalent cations are shown in Table 1. Although the maximum activity was 3.8-fold higher with Mg^2+^ than with Mn^2+^, the *K*_*0*.*5*_ for Mn^2+^ was 250-fold lower than the *K*_*0*.*5*_ for Mg^2+^, with no significant change in the constants for the substrates. This finding shows that Mn^2+^ is the preferred divalent cation, consistent with the geochemistry of the Archaean ocean [48]. The *K*_*0*.*5*_ for Mn^2+^ of *TpPK* (20 μM) is one of the smallest reported to date: the *K*_*0*.*5*_ for Mn^2+^ of the PK of *Thermoproteus tenax* is 800 μM, whereas the *K*_*0*.*5*_ for Mg^2+^ was the same for both enzymes (~5 mM) [10]. In addition, the PK of *Thermoproteus tenax* exhibited the same *V*_*max*_ (45 μmol min^-1^ mg^-1^) at 50°C in the presence of either Mn^2+^ or Mg^2+^ [10].

**Table 1:** Kinetic constants for *Tp*PK in the presence of Mg^2+^ and Mn^2+^.

	Mg^2+^		Mn^2+^	
	*K*_*m*_	*n*	*K*_*0*.*5*_	*n*
**PEP** ^**3-**^ **(mM)**	0.38 ± 0.03	___	1.0 ± 0.1	1.2 ± 0.1
**ADP- M** ^**2+**^ **(mM)**	0.16 ± 0.01	___	0.2 ± 0.02	1.2 ± 0.3
**M** ^**2+**^ **(mM)**	5.0 ± 0.28	1.8 ± 0.4	0.02 ± 0.001	1.6 ± 0.2
***V***_***max***_ **(μmol/ min • mg)**	183 ± 8		41 ± 1.9	

**Fig 2:**
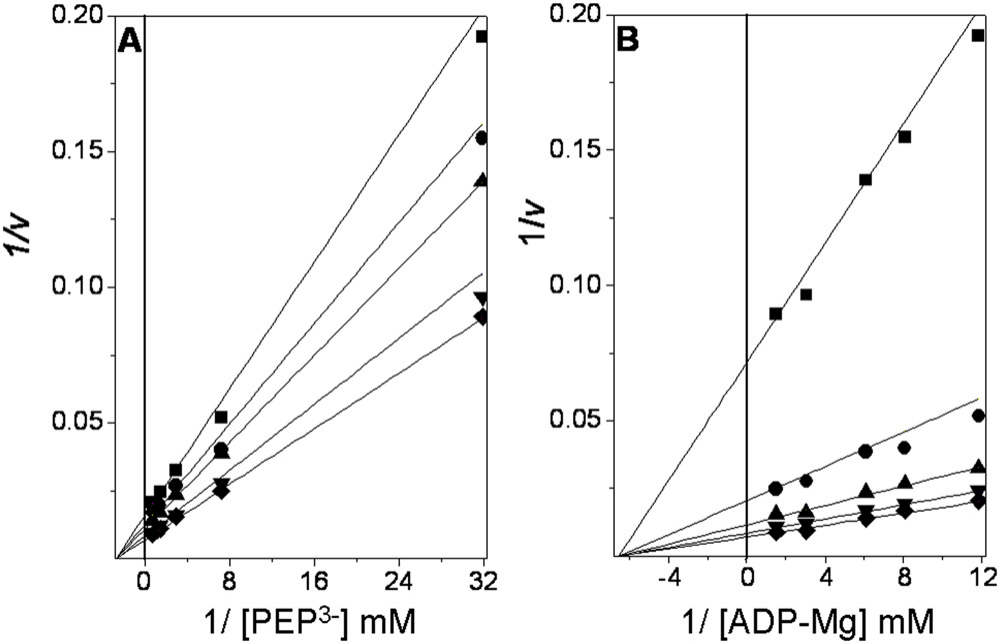
Double reciprocal plots from the initial velocity data of the *Tp*PK reaction. The reaction medium consisted of 3 ml of 50 mM Tris-HCl pH 6.0 containing 0.24 mM NADH, 30 mM Mg^2+^_free_, and 8 μg/ml LDH. The reciprocals of the concentrations of ionized PEP and ADP-Mg complexes are shown in the *abscissas* of each graph. **A**. The fixed concentrations of ADP-Mg were 0.084 (■), 0.12 (●), 0.16 (▲), 0.37 (▼), and 0.67 mM (♦). **B**. The fixed concentrations of PEP^3-^ were 0.031 (■), 0.14 (●), 0.34 (▲), 0.69(▼), and 1.30 mM (♦). The Mg^2+^_free_ concentration was kept constant at 30 mM. To maintain the ionic strength, (CH_3_)_4_NCl was added to a final salt concentration of 0.25 M. The reaction was started with the addition of PK. The concentrations of PK were 0.32 and 0.16 μg/ml for the three lowest and two highest substrate concentrations, respectively.

The data with Mg^2+^ from Fig. 2 were globally fit (nonlinear regression, Origin version 6.0) to the equation describing a rapid-equilibrium random-order mechanism: *v = V*_*max*_[A][B]/(*K*_*a*_ *K*_*b*_ *+ K*_*a*_ [B] + *K*_*b*_[A] + [A][B]), where *v* represents the initial velocity, A is PEP, B is ADP-Mg, and *K*_*a*_ and *K*_*b*_ are the Michaelis-Menten constants for PEP^3-^ and ADP-Mg, respectively. S.D. values are shown. The data (not shown) with Mn^2+^ were fitted to the Hill equation v = *V*_max_*[S]^*n*^/*K*_*0*.*5*_^*n*^+[S]^*n*^, where S indicates the substrate concentration of PEP^3-^, ADP-Mn or Mn^2+^. The mean and standard deviations of five experiments are shown.

### Effect of allosteric modulators

As for other K^+^-independent PKs, the binding of PEP and divalent cations to *Tp*PK was cooperative [10,11,40,41,42]. Therefore, we explored the effects of classic allosteric modulators, such as ribose-5-phosphate, AMP and fructose-1,6-bisphosphate on the binding of PEP on the response of this enzyme. In the presence of 2 mM ADP-Mn^2+^ and 0.2 mM Mn^2+^_free_, the addition of 0.5 mM ribose-5-phosphate increased the *V*_*max*_ by 41% with minor effects on the *K*_*0*.*5*_ for PEP and on the Hill number (S3A Fig. and S2 Table). In the presence of Mn^2+^, 10 mM fructose-1,6-bisphosphate increased the *K*_*0*.*5*_ for PEP 4-fold and increased the *V*_max_ by 34%, but it had no effect on the Hill number (S3B Fig. and S3 Table). In contrast, Mg^2+^ had no effect (not shown). AMP behaved as a competitive inhibitor of ADP-Mg with a *K*_*i*_ of 35 ± 1.2 mM, as described in the section on dead-end inhibition studies. In contrast with other PKs from hyperthermophilic archaea, which showed no response to classic allosteric effectors [10,11], *Tp*PK does exhibit discrete regulatory properties.

### Initial velocity of *Tp*PK

At a saturating concentration of K^+^, RMPK follows a random-order rapid equilibrium kinetic mechanism [49,50,51] that changes to an ordered mechanism with PEP as the first substrate in the absence of K^+^ [7]. In the absence of K^+^, the mutant E117K exhibits the same random-order rapid equilibrium kinetic mechanism [7]. These results indicate that in the muscle enzyme, the inner positive charge or K^+^ induces the closure of the active site and the arrangement of the residues involved in the binding of the nucleotide, allowing the random binding of PEP and ADP-Mg to the active site [7]. In this context, we were interested in determining the kinetic mechanism of *Tp*PK. The initial velocities of the reaction of *Tp*PK were measured without monovalent cations and with Mg^2+^ as the divalent cation. These experiments were carried out at various concentrations of one of the substrates and at fixed concentrations of the other (S4 Fig.). The double reciprocal plots of the initial velocities *versus* the ionized PEP concentrations intersected on the 1/S axis and to the left of the 1/*v* axis (Fig. 2A). When the concentration of ADP-Mg was varied, the lines intersected on the 1/S axis and to the left of the 1/*v* axis (Fig. 2B). This result indicates an ordered steady state or a random-order rapid equilibrium kinetic mechanism. The data were fit to the equation described in Table 1, and the kinetic constants obtained are summarized in Table 2.

**Table 2:** Intersecting patterns, kinetic mechanisms, and kinetic constants for *Tp*PK.

Initial velocity intersecting patterns		Kinetic mechanism	*V*_*max*_	*K*_*m*_ PEP^3-^	*K*_*m*_ ADP-Mg	*k*_*cat*_	Log *k_cat_/ K_m_* PEP^*3-*^	Log *k_cat_/ K_m_* ADP-Mg
1/*v vs*.1/PEP^3-^	1/*v vs*.1/ADP-Mg		μmol/min·mg	mM	mM	s^-1^	M^-1^ s^-1^	M^-1^ s^-1^
Intersects to the left of the 1/*v* axis and on the 1/S axis	Intersects to the left of the 1/*v* axis and on the 1/S axis	Random rapid Equilibrium	183 ± 8	0.38 ±0.03	0.16 ±0.01	626	6.22	6.59

Intersecting patterns were taken from the double reciprocal plots of the initial velocity data. The data shown in Fig. 2 were globally fit to the equation describing a rapid-equilibrium random-order mechanism as described in Table 1. The specificity coefficients *k*_cat_/*K*_*m*_ (M^-1^ s^-1^) are expressed in log form.

### Dead-end inhibition studies

**Fig 3:**
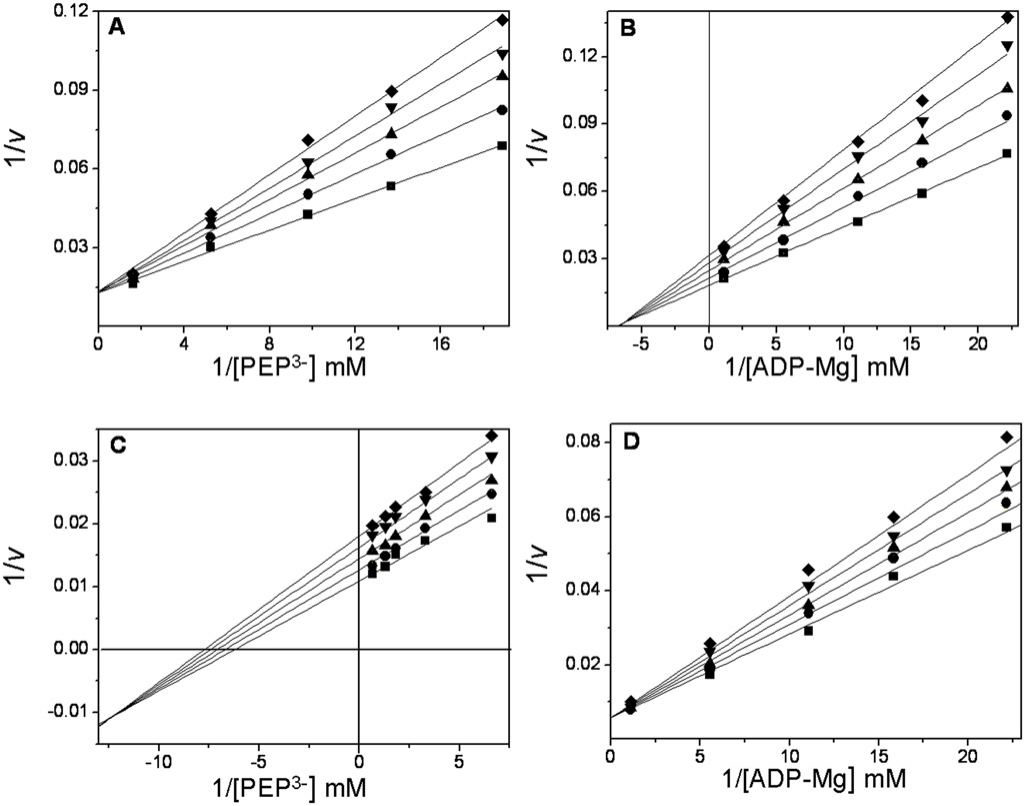
Dead-end inhibition patterns and inhibition constants for oxalate and AMP in *Tp*PK. The experimental conditions were as indicated in Fig. 2. The reciprocals of the concentrations of ionized PEP and ADP-Mg complexes are shown in the abscissas of each graph. In plot **A**, the concentrations of PEP^3-^ were 0.054, 0.077, 0.10, 0.22, and 1.1 mM. The Mg^2+^_free_ and ADP concentrations were kept constant at 30 mM and 2 mM, respectively. The fixed concentrations of oxalate were 0 (∎), 10 (●), 20 (▴), 30 (▾) and 40 (◆) μM. In plot **B**, the concentrations of the ADP-Mg complexes were 0.045, 0.063, 0.090, 0.18, and 0.90 mM. The ionized PEP concentration was kept constant at 30 mM. The Mg^2+^_free_ and oxalate concentrations were as in plot A. In plot **C**, the concentrations of PEP^3-^ were 0.15, 0.30, 0.55, 0.77, and 1.5 mM. The Mg^2+^_free_ and ADP concentrations were as in plot A. The fixed concentrations of AMP were 0 (∎), 4 (●), 8 (▴), 12 (▾) and (◆) 16 mM. In plot **D**, the concentrations of ADP were as in plot *B*. The Mg^2+^_free_ and ionized PEP concentrations were kept constant at 30 mM. The concentrations of AMP were 0 (∎), 8 (●), 12 (▴), 16(▾) and 20 (◆) mM.

Dead-end inhibitors are powerful tools for investigating the kinetic mechanisms of enzymes [52]. Here, oxalate and AMP were used as dead-end analogs of PEP [53] and of ADP, respectively. The patterns of oxalate inhibition *versus* ionized PEP and ADP-Mg were competitive (Fig. 3A) and noncompetitive (Fig. 3B), respectively. With AMP, the inhibition was mixed (Fig. 3C) and competitive with ionized PEP and ADP-Mg, respectively (Fig. 3D). The data were globally fit to the equations that describe linear competitive inhibition, linear non-competitive inhibition or linear mixed inhibition. The inhibition patterns and inhibition constants are shown in Table 3. Collectively, the data show that oxalate is a competitive inhibitor with respect to PEP, whereas AMP is a competitive inhibitor with respect to ADP-Mg. This indicates that the analogs and the substrates bind to the same site. Because oxalate is a non-competitive inhibitor with respect to ADP-Mg, it may be concluded that oxalate forms a non-productive ternary complex and thereby diminishes the *V*_*max*_, without altering the binding of ADP-Mg. The same argument holds for AMP with respect to PEP, except that the inhibition pattern observed (Fig. 3C) was a mixed-type pattern with α < 1 (factor affecting *K*_*i*_) for AMP inhibition *versus* PEP. This difference indicated that the enzyme-AMP binary complex has a higher affinity for PEP than the free enzyme. Therefore, the results obtained using dead-end inhibitors indicate that *Tp*PK follows a rapid-equilibrium random-order kinetic mechanism, as reported for RMPK in the presence of K^+^ [7,52,53,54] or the mutant E117K-PK in the absence of K^+^ [7]. This finding indicates that PEP and ADP-Mg bind independently to *Tp*PK despite the absence of an internal positive charge. Similarly, it has been shown that in the presence of 40% DMSO, RMPK follows a rapid-equilibrium random-order mechanism in the absence of K^+^ [7]. The activity of the enzyme under these conditions is 1000-fold higher than in aqueous medium without K^+^ [55]. Kinetic and spectroscopic studies have shown that in the absence of K^+^, 40% DMSO induces the acquisition of the active (closed lid) conformation of the enzyme [55,56]. Consequently, the positive internal charge provided by Lys cannot explain the K^+^-independent activity observed in RMPK with 40% DMSO or in *Tp*PK in fully aqueous media. Therefore, in accordance with the data for RMPK in DMSO and in the absence of K^+^, the data suggest that the overall conformation of *Tp*PK contributes to the catalytic activity.

**Table 3:** Dead-end inhibition patterns and inhibition constants for oxalate and AMP in *Tp*PK.

Dead-end analog of PEP^3-:^ oxalate		Dead-end analog of ADP-Mg: AMP		*K*_*i*_ (oxalate) mM	*K*_*i*_ (AMP) mM
1/*v vs*. 1/PEP, fixed ADP-Mg	1/*v vs*. 1/ADP-Mg, fixed PEP^3-^	1/*v vs*. 1/PEP^3-^, fixed ADP-Mg	1/*v vs*. 1/ ADP-Mg, fixed PEP^3-^		
C	NC	MT	C	0.05±0.002	35 ±1.2

Inhibition patterns were taken from the double reciprocal plots of the inhibition experiments (Fig. 3). Simple inhibition patterns were confirmed from linear replots of the slopes or intercepts *versus* the inhibitor concentrations (not shown). The inhibition constants were calculated from the fits of the complete data set to the corresponding equations for linear competitive inhibition (C) *v = V*[S]/(*K*_*m*_ (1+ [I]/*K*_*i*_) +[S]), linear noncompetitive inhibition (NC), or linear mixed inhibition (MT) *v = V*[S]/(*K*_*m*_ (1+ [I]/*K*_*i*_) + [S](1+[I]/α*K*_*i*_)), where α = 1 and α < 1 for NC and MT, respectively; *K*_*i*_ is the inhibition constant.

### Thermostability of *Tp*PK analyzed by DSC

**Fig 4:**
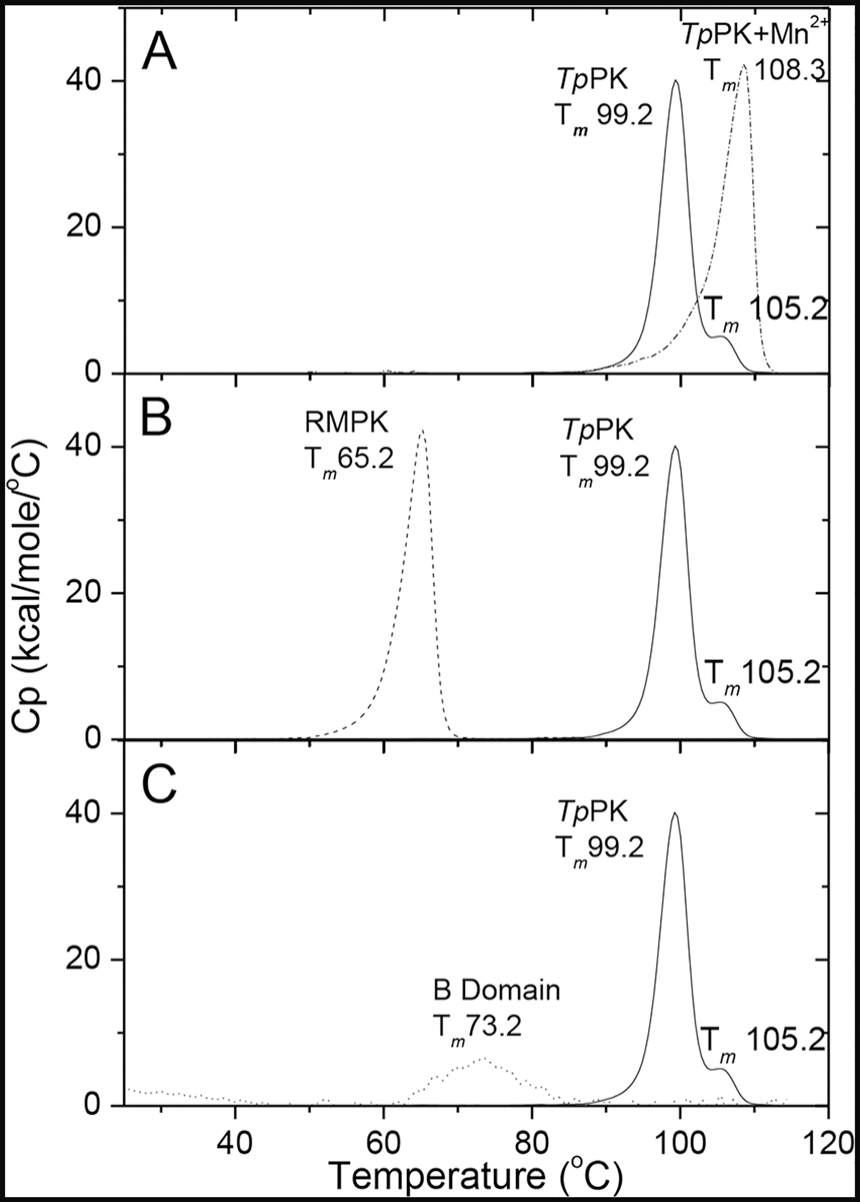
Differential scanning calorimetry of the *Tp*PK. **A**. *Tp*PK without and with 0.2 mM Mn^2+^ are represented by solid and dashed-dotted lines, respectively. **B**. *Tp*PK: solid line, RMPK: dashed line, **C**. *Tp*PK: solid line, the B domain: dotted line. The enzyme concentration was 1.0 mg/ml (19.47 μM monomer) for *Tp*PK, the B domain concentration was 0.18 mg/ml (19.55 μM of domain). The scan rate was 1.5°C/min.

Because *Tp*PK is a hyperthermophilic enzyme, we were interested in studying and comparing its thermal stability with the mesophilic RMPK. Thermal denaturation of *Tp*PK was studied by DSC at a rate of 2.5°C/min at various protein concentrations in the range of 0.1–2.0 mg/ml. The *T*_*m*_s were similar and independent of the protein concentration within the 10- to 20-fold range. This result strongly suggests that the dissociation of the subunits is not likely to be involved in the denaturation process, and no evident aggregation was observed within this protein concentration range. In all cases, the calorimetric transitions were irreversible, as demonstrated by the lack of a thermal effect in reheating runs. In addition, these transitions were also strongly dependent on the scanning rates (within the range of 0.5–2.5°C/min), indicating that the denaturation process of *Tp*PK is under kinetic control (data not shown). It is relevant to mention that under these experimental conditions, *Tp*PK exhibited two calorimetric transitions. Additional DSC experiments were conducted at 1 mg/ml and 1.5°C/min. In Fig. 4A, the thermograms for *Tp*PK with and without 0.2 mM MnCl_2_ are shown. The *T*_*m*_ values of the two transitions of *Tp*PK without Mn^2+^ were 99.2 and 105.2°C; with Mn^2+^, the *T*_*m*_ value increased to 108.4°C, and a single transition was observed. This result indicated that the enzyme was stabilized with Mn^2+^ and that the denaturation occurred in a single step. It is known that metal ions that bind with high affinity to specific sites often stabilize the conformation of proteins [57,58].

In Fig. 4B, the thermogram for the hyperthermophilic *Tp*PK was compared with that of the mesophile RMPK. In contrast to the two calorimetric transitions of *Tp*PK, RMPK only exhibited one transition with a *T*_*m*_ of 65°C. The two transitions observed in the thermogram of *Tp*PK suggested independent denaturation of its domains; whereas the thermogram of RMPK indicated a single global denaturation. Similar transitions were observed before or after removal of the His_6_ tag of recombinant *Tp*PK, ruling out the possibility that any of the transitions were due to the extra peptide (data not shown).

To assess whether the second transition corresponded to the B domain, this domain was cloned, overexpressed and purified as described in the Materials and Methods section. Fig. 4C shows that the *T*_*m*_ of the B domain was 73.5°C, i.e., very low compared to the second transition of *Tp*PK (105.2°C). It seems therefore that inter-domain interactions of *Tp*PK stabilize the B domain as reported for the *β*-1,4-glycanase from *Cellulomonas fimi* [59]. Therefore, the DSC of the isolated domain of *Tp*PK is not sufficient to define the nature of the 105°C transition. However, when a single isolated β-sheet barrel domain is stable, a single transition coincident with the second transition of a whole protein has been reported [59].

**Fig 5:**
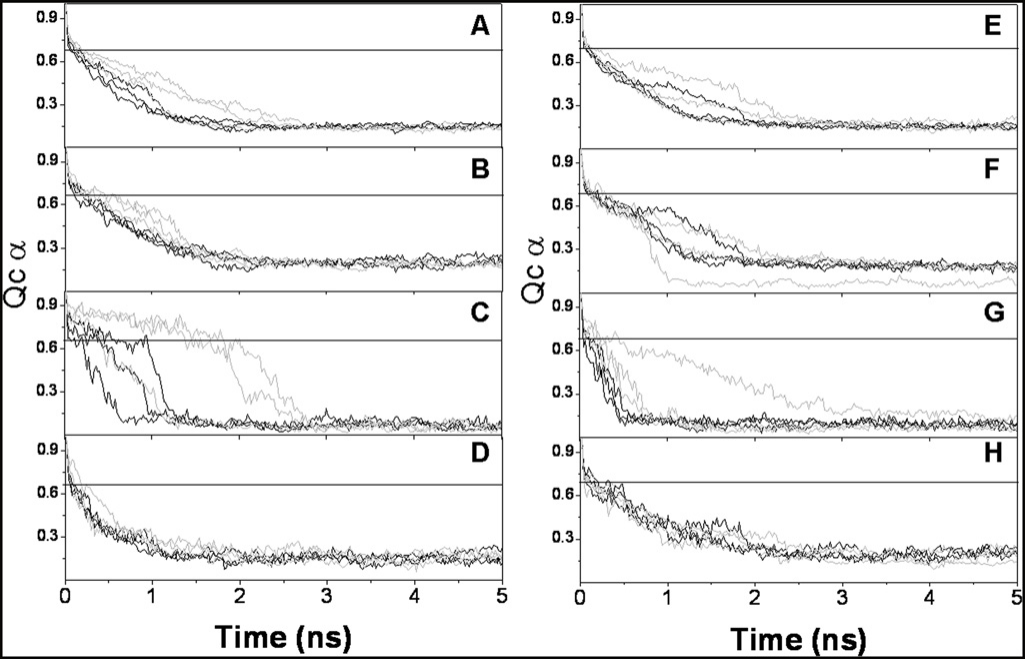
Loss of native contacts (Q_Cα_) *versus* time for the modeled *Tp*PKs and RMPKs at 500 K. The native contacts for the PK monomer and of the A, B and C domains are shown. *Tp*PKs and RMPKs correspond to panels **A** through **D** and to panels **E** through **H**, respectively. Simulations were performed for 50 ns in an implicit solvent at 500 K. The simulations were started from two different conformations of the enzymes, open (black lines) and closed (grey lines). The open and closed conformations for *Tp*PK were modeled as described in Material and Methods. The open and closed conformations for RMPK were obtained for PDBID 2G50 and PDBID 1A5U, respectively. The simulations were run in triplicate. Although the simulations were run for 10 to 50 ns, only the relevant periods for the transitions are shown.

Because the second 105.2°C transition could not be matched with that of the isolated B domain of *Tp*PK, this result could be attributed either to this domain or to any other rearrangement of the protein. Therefore, to gain insights on the origin of this second transition, a molecular dynamic simulation of a modeled monomer of *Tp*PK was carried out. Evaluation of the model quality is shown in S1 Table. In Fig. 5A, the time course for the first 5 ns of the 50 ns for the triplicate molecular dynamics at 500 K are shown for the monomers of *Tp*PK and RMPK. The Q values for *Tp*PK and RMPK were calculated for the whole monomer and for the A, B and C domains. The analysis of the native contacts within each domain of *Tp*PK *vs*. time indicated that the A and C domains lost 90% of their native contacts within 2–3 ns (Fig. 5B and 5D). In contrast, domain B retained a high Q value (~0.65) for up to 2.5 ns (Fig. 5C). It is noted that the most stable simulations of domain B of *Tp*PK were those that started from the closed structure. However, even those that started from the open structure were more stable than the RMPK counterpart. For RMPK, only one simulation showed a B domain reaching Q = 0.2 at approximately 2.5 ns. The rest were denatured before 1 ns. For reference, a value of Q = 0.7 is defined as the limit before a 60-residue helical protein leaves the native state to approach the molten globule state [60]. Further analysis of the denaturation process is shown in S5 Fig., in which the loss of secondary structure can be seen. It is clear from the simulations that at 500 K, the proteins are completely denatured. These results suggest that the second calorimetric transition of *Tp*PK is due to the thermal denaturation of the B domain, whereas RMPK was denatured in a single global event. Remarkably, *Tp*PK simulated at 300 K exhibited closure of the B domain over the A domain. The same phenomenon occurred in the three simulations. Under the same conditions, two of the three simulations of RMPK completed with an open cleft and one ended with a twisted lid over the A domain (S1 File https://docs.google.com/file/d/0B57RfHIF-7vbNG5KZDg0N0pIS28/edit?usp=drive_web).

### Comparative analysis of the structure of a mesophilic and a hyperthermophilic PK

Mesophilic PKs exhibit a high mobility of the B domain and are stabilized when the active site is partially or totally occupied. In this respect, Larsen *et al*. [61,38] determined the structure of RMPK in the presence of various ligands. The authors found that changes in the position of the B domain relative to the rest of the protein account for the various conformations. When the active site cleft is closed, the angle of reference is 0° and the active site is completely occupied by the complex K^+^-Mg^2+^-oxalate-ATP-Mg. When the active site is partially occupied by K^+^, Mg^2+^ and an analog of PEP, the B domain can exhibit different angles of rotation (11°, 21° or 41°) relative to the closed subunits, indicating that the closure of the active site varies from partially closed to totally open [61,38]. In the crystal structure of the PK apoenzyme from cat muscle, no electronic density was found for the B domain [62]. This result suggests that when the active site is empty, the mobile B domain cannot be modeled due to weak or absent electron density.

**Fig 6:**
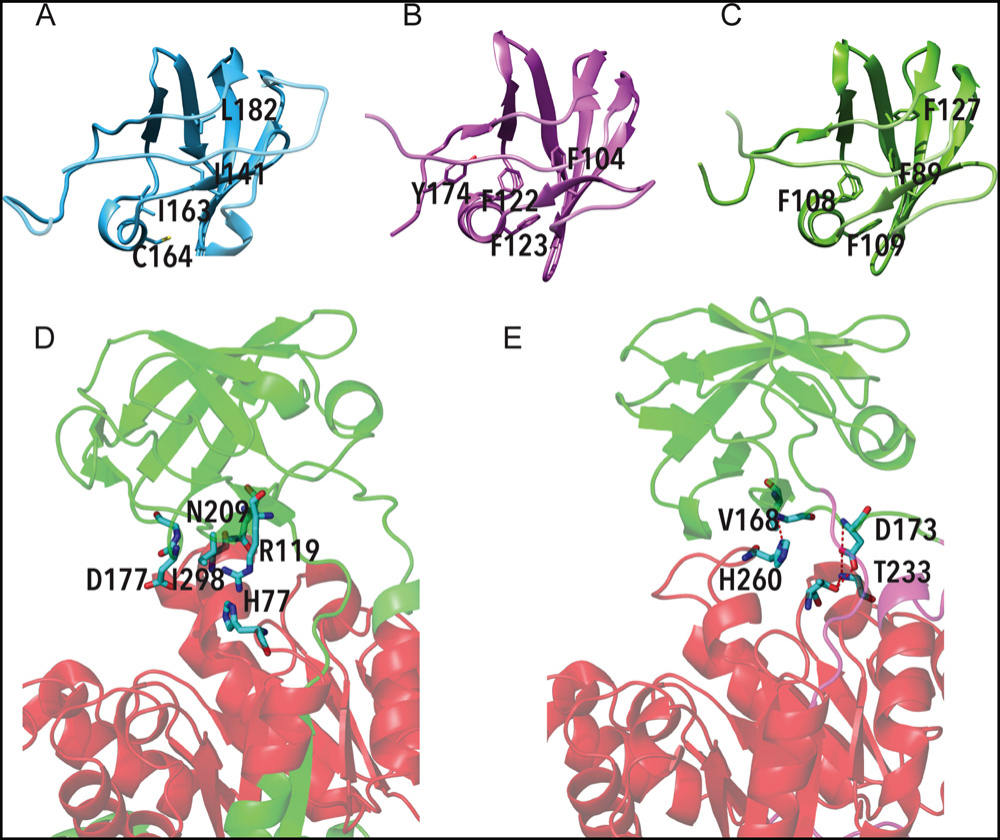
Models of the hydrophobic core of the B domain of RMPK (A), *Pa*PK (B) and *Tp*PK (C). Interactions between domains A and B of RMPK (D) and *Pa*PK (E). The aromatic residues at the hydrophobic core are represented as sticks. The residues involved in polar and hydrophobic interactions between domain A and domain B are shown as sticks. The red dotted lines highlight polar interactions. Notice that these interactions are absent in RMPK. This figure was constructed from the coordinates of RMPK and *Pa*PK deposited under file names 2G50 and 3QTG at the PDB. It is noteworthy that the interdomain interactions were not analyzed in the modeled monomer of *Tp*PK but were determined from the structure of *Pa*PK to obtain reliable results.

In contrast to the described cat muscle apo-PK structure, a complete electron density of the B domain of molecule A in the asymmetric unit was observed in the crystal structure of *Pa*PK (PDBID 3QTG), even though no ligands were present in the active site. This raises the question of how apo-*Pa*PK manages to conserve the complete electron density of the cleft in the absence of ligands that stabilize the B domain. To address this, the intra-domain and inter-domain interactions between the A and B domains of RMPK were compared with those of apo-*Pa*PK. More salt- bridges and beta-bridges were found in *Pa*PK than in RMPK (data not shown). The B domain of *Tp*PK was modeled and compared to those of *Pa*PK and RMPK (Fig. 6A- 6C). The hydrophobic core is formed by aromatic residues (Phe) in *Pa*PK as well as in the *Tp*PK model, which is in contrast to the aliphatic residues found in RMPK (Fig. 6B and 6C *vs*. 6A). Phe122, Phe123 and Phe104 of *Pa*PK correspond to Phe108, Phe109 and Phe89 of *Tp*PK and to Ile163, Cys164 and Ile141 of RMPK. It is worth mentioning that in Crenarchaeota, aromatic residues at these positions are highly conserved (positions 89, 108 and 109 are 85%, 88% and 100% conserved), whereas in RMPK, positions 163, 164 and 141 are substituted by aliphatic residues (S6 Fig.). The positions of the Phe residues observed in both *Pa*PK and *Tp*PK favor edge-to-face orientations of π-π electrostatic interactions (Table 4). To explore whether the π-π interactions of the B domain play a role in the closure of the lid over the A domain, the mutant F89I/F108I/F109C/F127L of *Tp*PK was modeled. In this mutant, we replaced the Phe residues present in *Tp*PK with those found in the corresponding positions of RMPK. Three simulations of this mutant were run at 300 K (S2 File https://drive.google.com/file/d/0B57RfHIF-7vbN1ZiQjNoS0RFbk0/view?usp=sharing). In contrast with the simulations of *Tp*PK, the open lid remained in two of the three simulations of the mutant *Tp*PK, while the third ended with a closed lid. Thus, the Phe-Phe interactions at this hydrophobic core likely contribute to the high stability and closed conformation of the B domain of *Tp*PK [63]. The interactions between domains A and B of RMPK and *Pa*PK are shown in 6D and 6E, respectively. Note that the interactions present in RMPK are different from those found in *Pa*PK. The interactions found in *Pa*PK are mostly in the hinges that join domains A and B, which allow a semi-closed cleft conformation, whereas RMPK does not have these interactions. Table 5 summarizes the atoms involved in the interactions and the distances between them. These two sets of distinct interactions, those within the B domain and those between domains A and B, may account for the putative high thermostability of the B domain of *Tp*PK. The high stability and low mobility of the active site cleft of the hyperthermophilic enzyme suggest that the structural arrangements could allow the catalytic activity of *Tp*PK in the absence of a positive charge. We are currently generating the constructs according to the structural analysis of the active site cleft to assess this working hypothesis.

**Table 4:** π-π interactions between phenylalanine pairs in the hydrophobic core of the B domain of pyruvate kinases from *Thermofilum pendens* (modeled) and from *Pyrobaculum aerophilum* (PDB ID 3QTG).

	Interaction between residues		Distance (Å)	Angle (grades)
***Tp*PK**	F89	F127	5.7	87.5
	F89	F108	5.6	51.7
	F108	F109	5.0	87.7
***Pa*PK**	F104	F122	5.9	32.7
	F122	F123	5.1	87.7
	F122	Y174	6.4	55.1

The distances were measured from the mass center, defined by the carbon atoms that form the aromatic rings. The angles were measured from the planes formed by the carbon atoms in each ring. Phe 89, 108 and 109 of *Tp*PK correspond to Phe 104, 122 and 123 of *Pa*PK and are 85%, 88% and 100% conserved in all Crenarchaeota phyla. Notice that position 109 in the enzyme from the Sulfolobales consists primarily of Tyr.

**Table 5:** Interactions between the A and B domains of RMPK (PDB ID 2G50) and of *Pa*PK (PDB ID 3QTG).

PDB	Donor	Acceptor	Distance (Å)	Contact type
2G50	I298-CG2	N209-CG	3.61	Hydrophobic
	I298-CG2	N209-CB	3.59	Hydrophobic
	I298-CG2	G178-CA	3.88	Hydrophobic
	I298-CD1	D177-C	3.52	Hydrophobic
	I298-CD1	D177-CA	3.72	Hydrophobic
	R119-CZ	H77-CE1	3.79	Hydrophobic
3QTG	G235-N	D173-OD2	2.98	Polar
	T233-OG1	D173-OD1	2.60	Polar
	G171-N	H260-ND1	3.03	Polar
	G235-N	D173-CG	3.62	Hydrophobic
	T233-CB	D173-CG	3.82	Hydrophobic
	H260-CE1	V168-CG1	3.51	Hydrophobic

The hinge regions, as determined by DynDom, were excluded from the analysis.

## Conclusions

*Tp*PK is encoded by a single-copy gene that is closely related to sequences from members of the Crenarchaeotal order Thermoproteales. These sequences are clustered in a group that includes PKs from the orders Desulfurococcales, Acidilobales, and Fervidicoccales, which contain amino acids other than Lys in the position corresponding to 117. Remarkably, *Tp*PK has Val70 at the corresponding position, and it does not require an internal positive charge near the active site for catalysis. Nevertheless, it follows a rapid-equilibrium random-order kinetic mechanism equal to that of RMPK in the presence of K^+^ and the E117K mutant in the absence of K^+^. These results indicate that in *Tp*PK, the closure of the active site and the arrangement of the residues involved in the binding of the nucleotide are independent of the internal positive charge and K^+^. The interactions (salt bridges, beta bridges, H^+^-bonds, hydrophobic and π-π interactions) present in the B domain of *Pa*PK and those modeled in *Tp*PK were analyzed and compared to those present in RMPK. The different interactions suggest that the B domain of the hyperthermophilic enzyme is highly stable. Taken together, the results lead to the working hypothesis that the structural arrangement of *Tp*PK allows catalysis by the enzyme in the absence of a positive charge.

## Supporting Information

S1 Fig Phylogenetic analysis of PKs and logos of selected residues located near the K^+^ binding site. Unrooted phylogenetic tree that includes all available PK protein sequences from the Archaea domain. Selected sequences from the Bacteria and the Eukarya domains were included as outer groups. Branches are colored according to the taxonomic group they belong. Logos showing conservation of the residues 113, 114, 117, and 120 (according to RMPK numbering) are shown adjacent to each taxonomic group included in the tree. The tree was inferred from 500 replicates, using the Maximum Likelihood method (18). The best tree with the highest log likelihood (-193785.3848) is shown. Similar trees were obtained with maximum-parsimony, minimum-evolution and neighbour-joining methods. The analysis involved 426 amino acid sequences (204 from archaea, 151 from bacteria and 71 from eukarya). The branches in the unrooted tree are drawn to scale, with the bar length indicating the number of substitutions per site. The proportion of replicate trees in which the associated taxa clustered together in a bootstrap test (500 replicates) is given next to selected branches.
(DOCX)

S2 Fig The SDS PAGE (A) and the Blue native PAGE (B) of the *Thermofilum pendens* pyruvate kinase. In (A) M indicates low molecular weight markers. In (B) the RMPK was used as molecular weight marker.
(DOCX)

S3 Fig Effect of different concentrations of Ribose-5-phosphate (A) and of Fructose- 1,6-bisphosphate (B) on the kinetics for PEP^3-^ of the *Tp*PK. The reaction mixture contained 50 mM Tris-HCl pH 6.0, 0.2 mM NADH, 0.1 mM Mn^2+^_free_, 3.0 mM ADP-Mn complex and 8 μg/ml LDH. In plot A the Ribose-5-phosphate concentrations were 0 (∎), 0.5 (●), 1 (▴) and 5 (▾). In plot B the fructose-1,6-bisphosphate concentrations were 0 (∎), 0.5 (●), 1 (▴), 5 (▾), and 10 mM (◆). The reaction was started by the addition of the PK. The amounts of the PK ranged from 0.15 to 1.2 μg/ml.
(DOCX)

S4 Fig Primary plots for PEP^3-^ (A) and ADP-Mg (B) of the *Tp*PK. The experimental conditions were those described in Fig. 2.
(DOCX)

S5 Fig Unfolding simulations (50ns) of the TpPK and the RMPK at 300, 400 and 500 K . The structural stability at the indicated temperatures is shown. The B domain is highlighted by a green square.
(DOCX)

S6 Fig Comparative logos of the amino acid residues located at the hydrophobic core of the B domain in Crenarchaeota and animals . The residues that comprise the hydrophobic core of the B domain, at position 89, 108, 109, and 127 (numbering according to the *Tp*PK), and equivalent positions in animals (numbering according to the RMPK) are showed.
(DOCX)

S1 File Simulations at 300 K of the RMPK and of a Model of the *Tp*PK. The movie shows a 3 by 3 animation where the 3 RMPK simulations at 300 K are on the top while the 3 *Tp*PK simulations at 300 K are at the bottom. All the simulations were run for 50 ns. The proteins are represented on the basis of the secondary structure and colored accordingly. PK480p.mov https://docs.google.com/file/d/0B57RfHIF-7vbNG5KZDg0N0pIS28/edit?usp = drive_web.
(DOCX)

S2 File Simulation at 300 K of the mutant (F89I/F108I/F109C/F127L) of a Model of the TpPK. The movie shows a triplicate animation of the mutant (F89I/F108I/F109C/F127L) of the *Tp*PK at 300 K. All the simulations were run for 50 ns. The proteins are represented on the basis of the secondary structure and colored accordingly. PKmutlid1080.mov https://drive.google.com/file/d/0B57RfHIF-7vbN1ZiQjNoS0RFbk0/view?usp=sharing.
(DOCX)

S1 Table Molprobity validation of the *Tp*PK model structure. (DOCX)

S2 Table Kinetic constants for PEP^3-^ at different concentrations of Ribose-5-phosphate. The data of S3A Fig. were fitted (nonlinear regression Origin version 6.0) to the Hill equation *v* = *V*_max_*[S]^*n*^/*K*_*0*.*5*_^*n*^+[S]^*n*^. The mean and standard deviation of five experiments are shown.
(DOCX)

S3 Table Kinetic constants for PEP3- at different concentrations of Fructose-1,6-bisphosphate . The data of S3B Fig. were fitted (nonlinear regression Origin version 6.0) to the Hill equation *v* = *V*_max_*[S]^*n*^/*K*_*0*.*5*_^*n*^+[S]^*n*^. The mean and standard deviation of five experiments are shown.
(DOCX)
