# Correction: New Insights on the Mechanism of the K^+^-Independent Activity of Crenarchaeota Pyruvate Kinases

**DOI:** 10.1371/journal.pone.0129757

**Published:** 2015-06-01

**Authors:** Gustavo De la Vega-Ruíz, Lenin Domínguez-Ramírez, Héctor Riveros-Rosas, Carlos Guerrero-Mendiola, Alfredo Torres-Larios, Gloria Hernández-Alcántara, José J. García-Trejo, Leticia Ramírez-Silva

There are errors in the Funding section. The complete, correct Funding information is as follows:

This work was supported by Dirección General de Apoyo al Personal Académico- Universidad Nacional Autónoma de México, Grants IN215912 (to L.R.-S.), IN216513 (to H.R.-R.), IA202714-2 (to G.H.-A.), IN201213 (to A.T.-L.), IN211012 (to J.J.G.-T.) and by Consejo Nacional de Ciencia y Tecnología Grant CB2011-164838 (to A.T.-L.) and CB2011-01-167622 (to J.J.G.-T.). The funders had no role in study design, data collection and analysis, decision to publish, or preparation of the manuscript.
