# Supplementary material for: New Insights on the Mechanism of the K+-Independent Activity of Crenarchaeota Pyruvate Kinases
Source: PLoS One. 2015 Mar 26;10(3):e0119233. doi: 10.1371/journal.pone.0119233 (PMC4374775; doi:10.1371/journal.pone.0119233)
Supplement: S1 Fig — Unrooted phylogenetic tree that includes all available PK protein sequences from the Archaea domain. Selected sequences from the Bacteria and the Eukarya domains were included as outer groups. Branches are colored according to the taxonomic group they belong. Logos showing conservation of the residues 113, 114, 117, and 120 (according to RMPK numbering) are shown adjacent to each taxonomic group included in the tree. The tree was inferred from 500 replicates, using the Maximum Likelihood method (18). The best tree with the highest log likelihood (-193785.3848) is shown. Similar trees were obtained with maximum-parsimony, minimum-evolution and neighbour-joining methods. The analysis involved 426 amino acid sequences (204 from archaea, 151 from bacteria and 71 from eukarya). The branches in the unrooted tree are drawn to scale, with the bar length indicating the number of substitutions per site. The proportion of replicate trees in which the associated taxa clustered together in a bootstrap test (500 replicates) is given next to selected branches. (DOCX) [file pone.0119233.s001.docx]

#
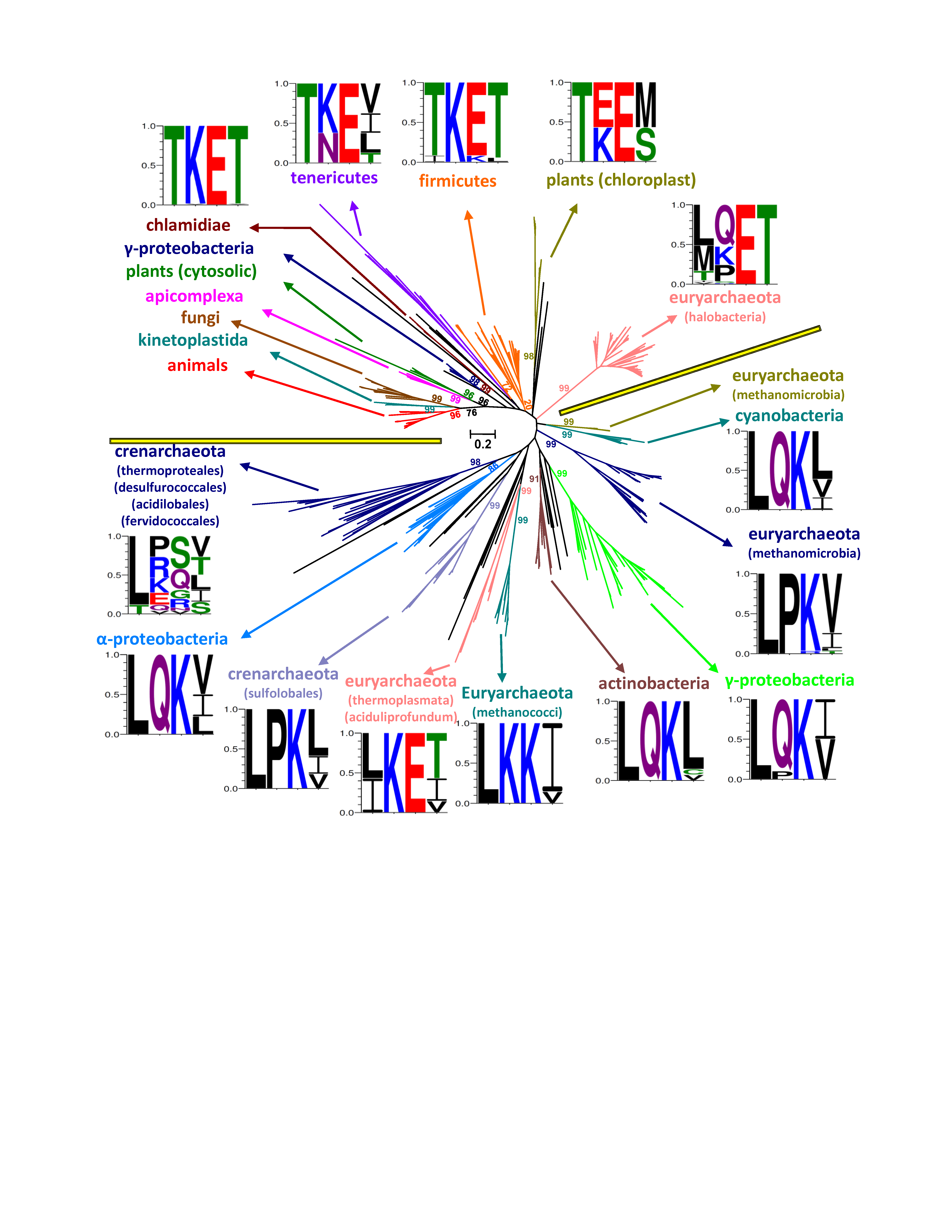


# S1 Figure. Phylogenetic analysis of PKs and logos of selected residues located near the K^+^ binding site. Unrooted phylogenetic tree that includes all available PK protein sequences from the Archaea domain. Selected sequences from the Bacteria and the Eukarya domains were included as outer groups. Branches are colored according to the taxonomic group they belong. Logos showing conservation of the residues 113, 114, 117, and 120 (according to RMPK numbering) are shown adjacent to each taxonomic group included in the tree. The tree was inferred from 500 replicates, using the Maximum Likelihood method (18). The best tree with the highest log likelihood (-193785.3848) is shown. Similar trees were obtained with maximum-parsimony, minimum-evolution and neighbour-joining methods. The analysis involved 426 amino acid sequences (204 from archaea, 151 from bacteria and 71 from eukarya). The branches in the unrooted tree are drawn to scale, with the bar length indicating the number of substitutions per site. The proportion of replicate trees in which the associated taxa clustered together in a bootstrap test (500 replicates) is given next to selected branches.
