# Supplementary material for: New Insights on the Mechanism of the K+-Independent Activity of Crenarchaeota Pyruvate Kinases
Source: PLoS One. 2015 Mar 26;10(3):e0119233. doi: 10.1371/journal.pone.0119233 (PMC4374775; doi:10.1371/journal.pone.0119233)
Supplement: S2 Fig — In (A) M indicates low molecular weight markers. In (B) the RMPK was used as molecular weight marker. (DOCX) [file pone.0119233.s002.docx]

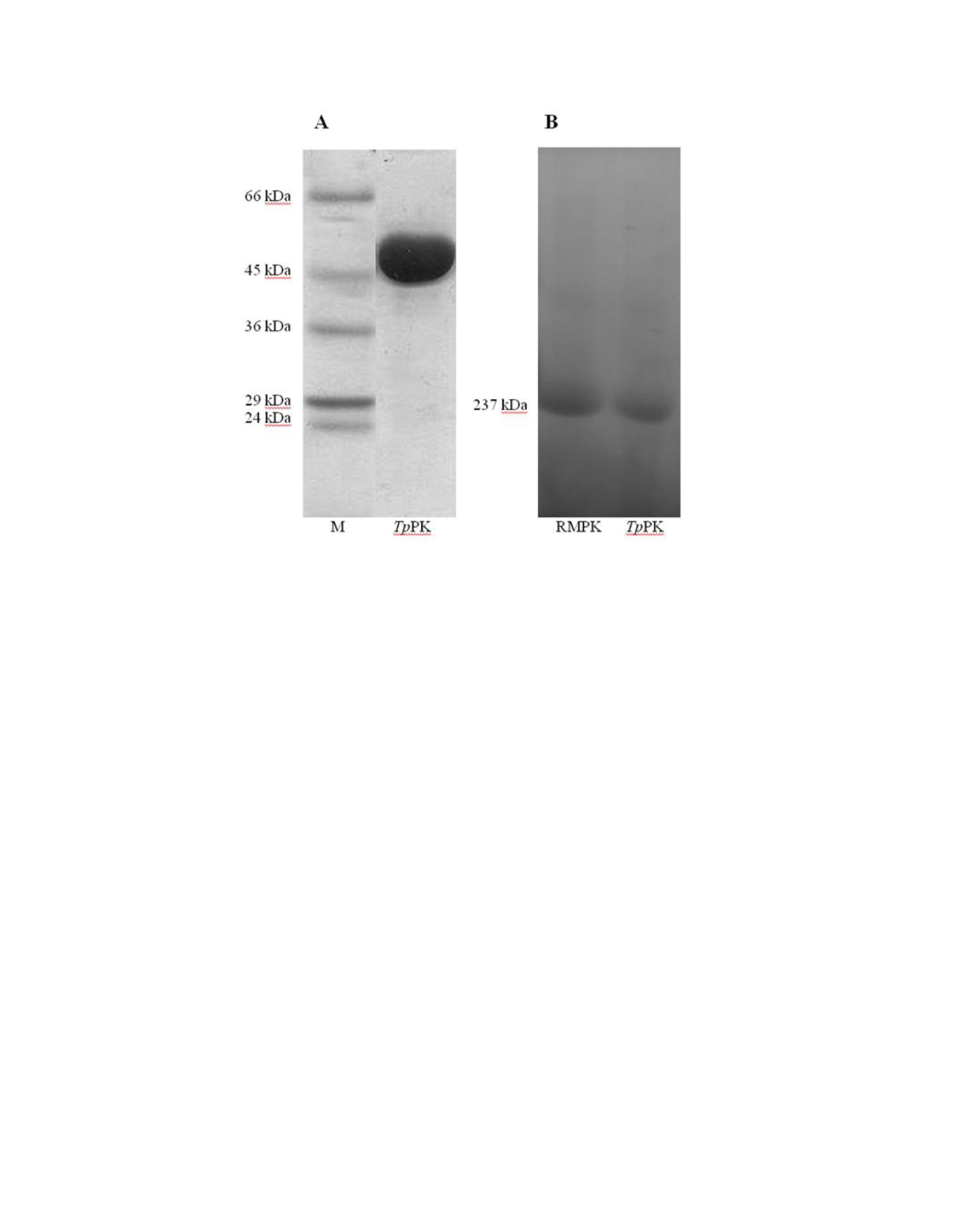


**S2 Figure. The SDS PAGE (A) and the Blue native PAGE (B) of the *Thermofilum pendens* pyruvate kinase.** In (A) M indicates low molecular weight markers. In (B) the RMPK was used as molecular weight marker.
