# Supplementary material for: New Insights on the Mechanism of the K+-Independent Activity of Crenarchaeota Pyruvate Kinases
Source: PLoS One. 2015 Mar 26;10(3):e0119233. doi: 10.1371/journal.pone.0119233 (PMC4374775; doi:10.1371/journal.pone.0119233)
Supplement: S3 Fig — The reaction mixture contained 50 mM Tris-HCl pH 6.0, 0.2 mM NADH, 0.1 mM Mn2+ free, 3.0 mM ADP-Mn complex and 8 μg/ml LDH. In plot A the Ribose-5-phosphate concentrations were 0 (∎), 0.5 (●), 1 (▴) and 5 (▾). In plot B the fructose-1,6-bisphosphate concentrations were 0 (∎), 0.5 (●), 1 (▴), 5 (▾), and 10 mM (◆). The reaction was started by the addition of the PK. The amounts of the PK ranged from 0.15 to 1.2 μg/ml. (DOCX) [file pone.0119233.s003.docx]

.

.

**S3 Figure. Effect of different concentrations of Ribose-5-phosphate (A) and of Fructose- 1,6-bisphosphate (B) on the kinetics for PEP^3-^ of the *Tp*PK.** The reaction mixture contained 50 mM Tris-HCl pH 6.0, 0.2 mM NADH, 0.1 mM Mn^2+^_free_ , 3.0 mM ADP-Mn complex and 8 μg/ml LDH. In plot A the Ribose-5-phosphate concentrations were 0 (■), 0.5 (●), 1 (▲) and 5 (▼). In plot B the fructose-1,6-bisphosphate concentrations were 0 (■), 0.5 (●), 1 (▲), 5 (▼), and 10 mM (♦). The reaction was started by the addition of the PK. The amounts of the PK ranged from 0.15 to 1.2 μg/ml.
