# Supplementary material for: New Insights on the Mechanism of the K+-Independent Activity of Crenarchaeota Pyruvate Kinases
Source: PLoS One. 2015 Mar 26;10(3):e0119233. doi: 10.1371/journal.pone.0119233 (PMC4374775; doi:10.1371/journal.pone.0119233)
Supplement: S4 Fig — The experimental conditions were those described in Fig. 2. (DOCX) [file pone.0119233.s004.docx]

**S4 Figure.** **Primary plots for PEP^3-^ (A) and ADP-Mg (B) of the *Tp*PK.** The experimental conditions were those described in Figure 2.
