# Supplementary material for: New Insights on the Mechanism of the K+-Independent Activity of Crenarchaeota Pyruvate Kinases
Source: PLoS One. 2015 Mar 26;10(3):e0119233. doi: 10.1371/journal.pone.0119233 (PMC4374775; doi:10.1371/journal.pone.0119233)
Supplement: S5 Fig — . The structural stability at the indicated temperatures is shown. The B domain is highlighted by a green square. (DOCX) [file pone.0119233.s005.docx]

#
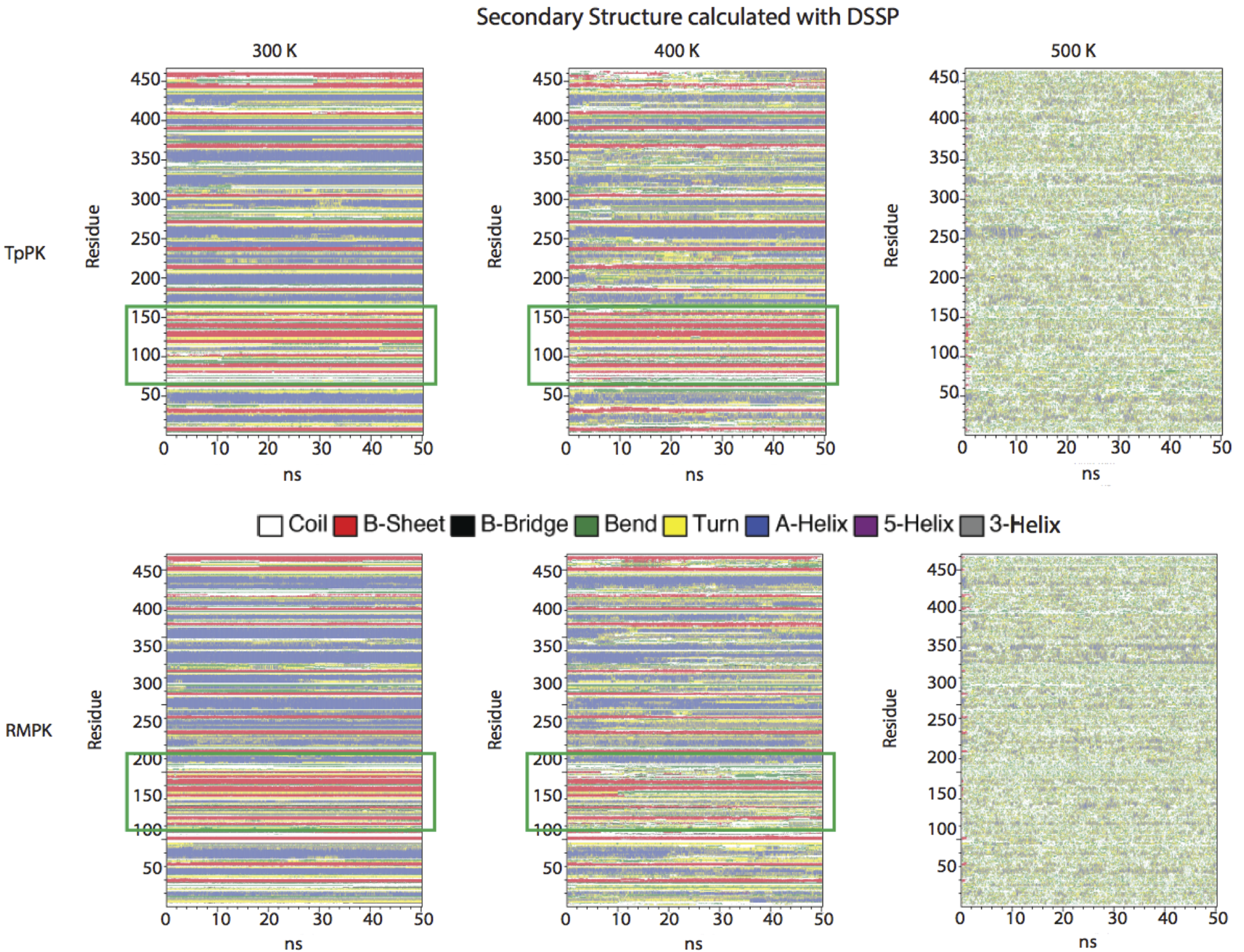


# S5 Figure. Unfolding simulations (50ns) of the *Tp*PK and the RMPK at 300, 400 and 500 K. The structural stability at the indicated temperatures is shown. The B domain is highlighted by a green square.
