# Supplementary material for: New Insights on the Mechanism of the K+-Independent Activity of Crenarchaeota Pyruvate Kinases
Source: PLoS One. 2015 Mar 26;10(3):e0119233. doi: 10.1371/journal.pone.0119233 (PMC4374775; doi:10.1371/journal.pone.0119233)
Supplement: S6 Fig — . The residues that comprise the hydrophobic core of the B domain, at position 89, 108, 109, and 127 (numbering according to the TpPK), and equivalent positions in animals (numbering according to the RMPK) are showed. (DOCX) [file pone.0119233.s006.docx]

#

# S6 Figure. Comparative logos of the amino acid residues located at the hydrophobic core of the B domain in Crenarchaeota and animals. The residues that comprise the hydrophobic core of the B domain, at position 89, 108, 109, and 127 (numbering according to the *Tp*PK), and equivalent positions in animals (numbering according to the RMPK) are showed.
