# Supplementary material for: New Insights on the Mechanism of the K+-Independent Activity of Crenarchaeota Pyruvate Kinases
Source: PLoS One. 2015 Mar 26;10(3):e0119233. doi: 10.1371/journal.pone.0119233 (PMC4374775; doi:10.1371/journal.pone.0119233)
Supplement: S1 File — The movie shows a 3 by 3 animation where the 3 RMPK simulations at 300 K are on the top while the 3 TpPK simulations at 300 K are at the bottom. All the simulations were run for 50 ns. The proteins are represented on the basis of the secondary structure and colored accordingly. PK480p.mov https://docs.google.com/file/d/0B57RfHIF-7vbNG5KZDg0N0pIS28/edit?usp = drive_web. (DOCX) [file pone.0119233.s007.docx]

S1 Video

PK480p.mov [https://docs.google.com/file/d/0B57RfHIF-7vbNG5KZDg0N0pIS28/edit?usp=drive_web](https://docs.google.com/file/d/0B57RfHIF-7vbNG5KZDg0N0pIS28/edit?usp=drive_web)

**Simulations at 300 K of the RMPK and of a Model of the *Tp*PK.** The movie shows a 3 by 3 animation where the 3 RMPK simulations at 300 K are on the top while the 3 *Tp*PK simulations at 300 K are at the bottom. All the simulations were run for 50 ns. The proteins are represented on the basis of the secondary structure and colored accordingly.
