# Supplementary material for: New Insights on the Mechanism of the K+-Independent Activity of Crenarchaeota Pyruvate Kinases
Source: PLoS One. 2015 Mar 26;10(3):e0119233. doi: 10.1371/journal.pone.0119233 (PMC4374775; doi:10.1371/journal.pone.0119233)
Supplement: S2 File — The movie shows a triplicate animation of the mutant (F89I/F108I/F109C/F127L) of the TpPK at 300 K. All the simulations were run for 50 ns. The proteins are represented on the basis of the secondary structure and colored accordingly. PKmutlid1080.mov https://drive.google.com/file/d/0B57RfHIF-7vbN1ZiQjNoS0RFbk0/view?usp=sharing. (DOCX) [file pone.0119233.s008.docx]

S2 Video

PKmutlid1080.mov <https://drive.google.com/file/d/0B57RfHIF-7vbN1ZiQjNoS0RFbk0/view?usp=sharing>

**Simulation at 300 K of the mutant (F89I/F108I/F109C/F127L) of a Model of the TpPK.**  The movie shows a triplicate animation of the mutant (F89I/F108I/F109C/F127L) of the *Tp*PK at 300 K. All the simulations were run for 50 ns. The proteins are represented on the basis of the secondary structure and colored accordingly.
