# Supplementary material for: New Insights on the Mechanism of the K+-Independent Activity of Crenarchaeota Pyruvate Kinases
Source: PLoS One. 2015 Mar 26;10(3):e0119233. doi: 10.1371/journal.pone.0119233 (PMC4374775; doi:10.1371/journal.pone.0119233)
Supplement: S1 Table — (DOCX) [file pone.0119233.s009.docx]

| **S1 Table. Molprobity validation of the *Tp*PK model structure** | | | |
| --- | --- | --- | --- |
| All-Atom | Clashscore, all atoms | 0 | 100^th^ percentile |
| Contacts |  |  |  |
|  | Poor rotamers | 0 |  |
| Protein | Ramachandran outliers | 0.9 % |  |
| Geometry | Ramachandran favored | 99.1 % |  |
|  | Molprobity score | 1.65 | 91^st^ percentile |
