# Supplementary material for: New Insights on the Mechanism of the K+-Independent Activity of Crenarchaeota Pyruvate Kinases
Source: PLoS One. 2015 Mar 26;10(3):e0119233. doi: 10.1371/journal.pone.0119233 (PMC4374775; doi:10.1371/journal.pone.0119233)
Supplement: S2 Table — The data of S3A Fig. were fitted (nonlinear regression Origin version 6.0) to the Hill equation v = V max*[S]n/K 0.5 n+[S]n. The mean and standard deviation of five experiments are shown. (DOCX) [file pone.0119233.s010.docx]

**S2 Table. Kinetic constants for PEP^3-^ at different concentrations of Ribose-5-phosphate.**

| **Rib-5-P**  **(mM)** | ***V*_max_**  **(μmol/min.mg)** | ***K_0.5_***  **(mM)** | ***n*** |
| --- | --- | --- | --- |
| 0 | 41 ± 2 | 0.95 ± 0.11 | 1.2 ± 0.11 |
| 0.5 | 58 ± 4 | 1.94 ± 0.3 | 1.1 ± 0.08 |
| 1 | 54 ± 4 | 1.81 ± 0.3 | 1.12 ± 0.9 |
| 5 | 39 ± 2 | 1.21 ± 0.2 | 1.83 ± 0.3 |

The data of Fig. S3A were fitted (nonlinear regression Origin version 6.0) to the Hill equation *v*=*V_max_**[S]*^n^*/*K_0.5_^n^*+[S]*^n^.* The mean and standard deviation of five experiments are shown.
