# Supplementary material for: New Insights on the Mechanism of the K+-Independent Activity of Crenarchaeota Pyruvate Kinases
Source: PLoS One. 2015 Mar 26;10(3):e0119233. doi: 10.1371/journal.pone.0119233 (PMC4374775; doi:10.1371/journal.pone.0119233)
Supplement: S3 Table — . The data of S3B Fig. were fitted (nonlinear regression Origin version 6.0) to the Hill equation v = V max*[S]n/K 0.5 n+[S]n. The mean and standard deviation of five experiments are shown. (DOCX) [file pone.0119233.s011.docx]

**S3 Table. Kinetic constants for PEP^3-^ at different concentrations of Fructose-1,6-bisphosphate.**

| **Fru-1,6-BP**  **(mM)** | ***V*_max_**  **(μmol/min.mg)** | ***K_0.5_***  **(mM)** | ***n*** |
| --- | --- | --- | --- |
| 0 | 41 ± 2 | 0.95 ± 0.11 | 1.2 ± 0.11 |
| 0.5 | 47 ± 2 | 0.96 ± 0.08 | 1.1 ± 0.06 |
| 1 | 53 ± 5 | 1.5 ± 0.36 | 0.96 ± 0.11 |
| 5 | 61 ± 7 | 3.9 ± 1.1 | 0.86 ± 0.06 |
| 10 | 55 ± 6 | 4 ± 1.1 | 0.97 ± 0.1 |

The data of Fig. S3B were fitted (nonlinear regression Origin version 6.0) to the Hill equation *v*=*V_max_**[S]*^n^*/*K_0.5_^n^*+[S]*^n^.* The mean and standard deviation of five experiments are

shown.
